# Development and validation of a knowledge, attitudes and practices questionnaire in the dietary management of irritable bowel syndrome

**DOI:** 10.1038/s41430-023-01306-7

**Published:** 2023-07-12

**Authors:** Katerina Belogianni, Paul Townsend Seed, Miranda Clare Elizabeth Lomer

**Affiliations:** 1 Faculty of Life Sciences and Medicine, Nutritional Sciences, King’s College London, 150 Stamford Street, London, SE1 9NH UK; 2 Faculty of Life Course and Population Sciences, Department of Women and Children’s Health, King’s College London, St Thomas’ Hospital, London, SE1 7EH UK; 3 Department of Nutrition and Dietetics, Guy’s and St Thomas’ NHS Foundation Trust, London, SE1 7EH UK

**Keywords:** Irritable bowel syndrome, Translational research

## Abstract

**Objective:**

To develop and validate a questionnaire assessing knowledge, attitudes and practices in the dietary management of IBS.

**Subjects/Methods:**

An initial pool of 151 questions was generated addressing three domains (knowledge, attitudes, practices). Academic/senior clinical dietitians (*n* = 5) provided written feedback and a focus group (*n* = 4 gastroenterology dietitians) was undertaken to evaluate content and face validity of the question-items. Items considered irrelevant were removed and the refined questionnaire was administered to dietitians with different levels of IBS experience (*n* = 154) for further psychometric testing. Item reduction analysis was assessed by item difficulty index, discrimination index and point-biserial correlation. Construct validity was assessed via principal component analysis (PCA) and the ‘known-groups’ method. Internal reliability was assessed by Kuder–Richarson Formula 20 and Cronbach’s alpha coefficient and external reliability by interclass correlation coefficient among participants who completed the instrument at baseline and two weeks later (*n* = 28).

**Results:**

Face and content validity resulted in the removal of 61 items from the initial 151 items. Psychometric testing was applied to the refined 90-item questionnaire administered to participating dietitians, resulting in the final 46-item questionnaire. Six factors were extracted by PCA with varimax rotation explaining 59.2% of the total variance. Partial confirmatory factor analysis showed an acceptable model fit (χ2/df = 2.11, CFI = 0.97, TLI = 0.96, RMSEA = 0.08, SRMR = 0.05). Significant differences were found in sum scores among dietitians with different levels of IBS experience. Internal reliability was >0.7 for each factor. External reliability was >0.6 for each factor and >0.7 for overall items of each domain.

**Conclusion:**

A validated questionnaire to use in practice and research to assess knowledge, attitudes and practices in the dietary management of IBS has been developed.

## Introduction

Irritable bowel syndrome (IBS) is a chronic and relapsing functional gastrointestinal disorder characterised by recurrent abdominal pain, bloating, flatulence and changes in bowel habits [1]. It is diagnosed using the Rome IV criteria and investigations are carried out (e.g., negative coeliac antibodies, normal faecal calprotectin), to exclude any organic disease with similar symptoms (e.g., coeliac disease, inflammatory bowel disease, cancer) [2]. The global prevalence of IBS varies between and within countries and has been reported from as low as 1.1% in France and Iran to 36% in Mexico [3]. In the UK, it affects at least 12% of the population [4]. Its’ pathogenesis is not fully elucidated but involves a complex and altered interaction between the gut-brain axis and biological factors [5]. IBS-related symptoms significantly decrease health-related quality of life, have societal consequences (e.g., isolation, work absence) and impose a profound burden on individuals and the healthcare system [4, 5].

NICE guidelines recommend lifestyle and dietary modifications, medication and psychological support (e.g., cognitive behavioural therapy) for the management of IBS [6]. First line lifestyle and dietary modifications include regular meals, adequate fluids, reduced intake of fat, caffeine and alcohol if associated with symptom generation, consideration of fibre intake, regular exercise and probiotic supplementation [6, 7]. Medication (e.g., antispasmodics, laxatives) can also be used alongside lifestyle modifications to relief symptoms. Many people will benefit by simple lifestyle modifications. Healthy eating and lifestyle recommendations can be provided by many healthcare practitioners involved in IBS management such as general practitioners, nurses, gastroenterologists and dietitians [7]. When patients report that specific foods exacerbate their symptoms, particularly diarrhoea, patients may be referred for second-line approaches in diet (low FODMAP diet) [6–8]. A diet restrictive in Fermentable Oligosaccharides Disaccharides Monosaccharides and Polyols (FODMAPs) is implemented in three distinctive stages, namely FODMAP restriction, FODMAP reintroduction, and FODMAP personalisation stage and its complexity entails nutritional risks [8]. FODMAP restriction reduces diet quality [9] and has been shown to negatively impact dietary fibre and calcium intakes [9, 10]. Furthermore, it consistently results in a reduction in the abundance of gastrointestinal Bifidobacteria [11]. Nutritional risks are reduced when people follow the diet under dietetic guidance and monitoring [12].

According to the World Health Organisation [13], the lack of knowledge and training of healthcare providers can negatively impact patients’ adherence to an intervention. As a result, assessing clinicians’ knowledge and competence to deliver dietary advice in IBS management can benefit patients. Some knowledge, attitudes and practices tools are based on Ajzen’s Theory of Planned Behaviour, which states that individuals’ knowledge will affect their attitudes and mediate their actual behaviour [14]. In dietetics, knowledge is the level of understanding of a topic, including the ability to remember and recall definitions and facts [15]. Attitudes include the perceptions on a topic that can positively or negatively affect a practice behaviour. Examples include self-efficacy or self-confidence to deliver dietary recommendations, perceived benefits that someone would gain from a dietary treatment or readiness to change and adopt a new practice. Practice refers to actions and recommendations in relation to the dietary management of a condition [15].

To the best of our knowledge, there is no existing validated questionnaire to assess knowledge, attitudes and practices in the dietary management of IBS. Parmenter & Wardle [16] stated that developing nutrition-related questionnaires of unknown reliability and validity, particularly de novo questionnaires, increases the risk of assessing a different construct of the targeted one. Therefore, a specific guidance should be followed to assess that the developed questionnaire is reliable, valid and suitable for the targeted population.

The aim of this research was to develop and validate a self-administered questionnaire to assess knowledge, attitudes and practices of dietitians in IBS management. In particular, the objectives of this research were to develop the content of a questionnaire for each domain (knowledge, attitudes, practices) and apply psychometric tests to establish its validity and reliability.

## Methods

A standardised methodology was followed to develop and evaluate the questionnaire [17]. These included the assessment of content validity, face validity and construct validity and the assessment of internal consistency and test-retest (external) reliability. The study was approved by the King’s College London Ethics Committee (17474).

### Item generation

An initial literature review in journal databases (MEDLINE, SCOPUS) was undertaken using appropriate keywords (diet, nutrition, FODMAPs, irritable bowel syndrome) to identify up to date information in relation to epidemiology, aetiopathogenesis and dietary treatment of the condition and confirm there were no similar existing questionnaires in the field. An initial pool of 151 questions was generated by a team of one academic and two clinical dietitians with expertise in the field, in addition to a senior clinical and academic dietitian who leads post-registration training courses in IBS dietary management in the UK. The initial pool of items included questions about epidemiology, aetiopathogenesis, diagnosis and medications used in IBS (25 items); gut microbiome and probiotics (21 items); bile acid malabsorption (16 items); FODMAP mechanisms and FOMDAP content of foods (34 items); first-line dietary advice (24 items); second-line dietary advice (12 items) and the low FODMAP diet (resources, explanation, education) (19 items). The FAO [15] manual was followed to develop the initial pool of items using straightforward (e.g., multiple-choice), dichotomous (True/False) and Likert scales as responses [18]. For the multiple-choice and true/false items, choosing the correct answer was given one point. Any incorrect or “not sure” answer was given zero points. For the Likert-scale items, the following points were given: strongly agree = 5, agree = 4, not sure = 3, disagree = 2 and strongly disagree = 1. For the negatively phrased items, scores were reversed (strongly disagree = 5, disagree = 4, not sure = 3, agree = 2 and strongly agree = 1). For the Practice items the following scores were applied: not applicable = 0, never = 1, sometimes = 2, often = 3, always = 4. For the negatively phrased items, scores were reversed (not applicable = 0, never = 4, sometimes = 3, often = 2, always = 1). Points were added together and a higher score indicated a higher performance in all sections.

### Content and face validity

To evaluate content validity, one academic staff and four senior clinical dietitians in gastroenterology reviewed the question-items and provided written comments with regards to content relevance, representativeness and technical quality of items in each domain. To evaluate face validity, a focus group with four dietitians working with patients with functional bowel disorders was conducted to discuss clarity, comprehension, layout, and appropriateness of the question-items.

### Sample size

Two approaches were used to determine the minimum sample size required: enough subjects for a valid factor analysis and enough subjects for a sound demonstration of test-rest reliability. According to Mundfrom et al. [19] in order to conduct factor analysis 130 subjects are needed for excellent agreement between the estimated factor structure and the true underlying factor. To perform the test-rest reliability analysis, sample size calculation was based on 1-way random effects analysis of variance model at 80% power, including a minimum acceptable reliability at 0.6 and a targeted reliability at 0.85 [20]. As a result, a sample size of at least 130 subjects was considered sufficient for the factor analysis and a sample of 28 participants for the external reliability analysis (intraclass correlation coefficient).

### Study participants and procedure

A convenience sampling strategy was used and the questionnaire was disseminated via social media platforms and dietetic networks. Eligible participants were registered or student dietitians with varying levels of experience in the dietetic management of IBS (i.e., none to expert). No age or other limitations were applied. The questionnaire was administered in an online survey using the Qualtrics Research Core^TM^ (www.qualtrics.com). Written consent was obtained before starting the survey. Participants were invited to complete the questionnaire at baseline and two weeks later to assess its’ external reliability. Demographic (age, sex, place and country of work) as well as information about participants’ experience working with IBS (e.g., years of experience, post-registration training, perceived competence in IBS management) were collected as part of the survey.

### Data analyses

#### Item reduction and scale evaluation

Item discrimination and item difficulty were measured for the multiple-choice items. Item discrimination was measured using Kelley’s formula [21] and Point-Biserial (Pearson) correlation coefficient [22] and items with values less than 0.4 were considered for removal. Item difficulty was calculated by dividing the total number of responses to the number of correct responses and very easy (>0.7) or very difficult (<0.3) items were considered for removal [23]. Distractor efficiency analysis was further performed and multiple-choice items with distractors selected by less than 5% of participants were considered for replacement or removal.

Exploratory factor analysis (EFA) with principal component analysis and varimax rotation was performed to explore the number of factors of the set of items and the variance explained by the factor model [24]. The criteria of the Bartlett’s test of sphericity (*p* < 0.001) and the Kaiser-Meyer-Olkin (KMO) measurement of sampling adequacy (>0.6) were met for a satisfactory factor analysis. The number of factors were determined using a scree plot and eigenvalues >1 and items with factor loadings below 0.4 were removed [25]. Labels were given to the identified factors. Exploratory factor analysis was followed by (partial) confirmatory factor analysis to test whether the proposed model was an acceptable model fit by fulfilling the following criteria: chi-square test of exact fit (χ2/df < 2), Comparative Fit Index (CFI) > 0.9, Tucker Lewis Index (TLI) > 0.9, Root Mean Square Error of Approximation (RMSEA) < 0.08 and Standardised Root Mean Square Residual (SRMR) < 0.08 [17, 26].

Internal reliability was determined by corrected item-total correlations, with acceptable levels ≥0.2 [27]. The Kuder–Richarson Formula 20 (KR20) was calculated for the multiple-choice items and the Cronbach’s alpha reliability coefficient (a) for the Likert-scale items with values > 0.6 indicating an acceptable and >0.7 a very good level of internal reliability [28]. Intra-class correlation coefficient (ICC) was conducted for test-retest (external) reliability to assess the consistency of sum scores across time with values > 0.5 indicating a moderate, >0.7 indicating good and >0.9 indicating excellent reliability [29]. Repeat participants were excluded from the analysis if they had attended any related diet and IBS course within the two weeks interval.

Construct validity was further established using the differentiation by ‘known-group’ method. This compared the performance of dietitians according to their experience in IBS management. For the analysis, a new variable was created to group participants (i.e., those with low, moderate and high experience in IBS) based on their responses to the questions “*How many years have you been working with IBS patients?”* and “*Have you had any post-registration training on the low FODMAP diet?”* The following criteria were used: no experience of IBS (e.g., students) or with ≤four years post-registration experience of IBS and no post-registration training (i.e. on the low FODMAP diet) (low); with ≤four years post-registration experience of IBS and undertaking/attended post-registration training or >four years post-registration experience of IBS without training or enroled/undertaking post-registration training (moderate); with >5 years of experience of IBS and post-registration training (high).

#### Statistical analyses

Data were analysed using SPSS 26.0 software. Data was entered, cleaned and checked before data analysis. Median and IQR were calculated for continuous variables, frequencies and percentages for categorical variables. Cronbach’s alpha coefficient was computed for internal consistency reliability, and intraclass correlation coefficient (ICC) was used to evaluate test-retest reliability. Construct validity was examined by exploratory factor analysis (EFA) and confirmatory factor analysis (CFA) after assessing sample adequacy and Bartlett’s test of sphericity using the aforementioned criteria. In the ‘known-group’ methods, the nonparametric Kruskal–Wallis H test (one-way ANOVA) was used to compare the sum scores of each factor as well as of overall knowledge, attitudes and practice items among the three groups. *P*-values < 0.05 were considered statistically significant.

## Results

The face and content validity analysis resulted in the removal of 61 items from the initial pool of 151 items based on the feedback received. Main feedback included the re-wording of some attitude items, concerns regarding the technicality, irrelevance and difficulty of some items (for example on bile acid malabsorption) and suggestions to avoid items without clear clinical recommendations for practice. The average time to complete the refined questionnaire was 20 min.

One hundred and eighty-eight participants consented to complete the administered 90-item questionnaire of which 154 dietitians provided complete answers and were included in the analysis (Table 1). The item reduction analysis showed that 12 items had an item discrimination index less than 0.4, 23 items had a difficulty index <0.3 or >0.7, 13 items had a point-biserial correlation coefficient <0.4 and 13 items had a total-item correlation <0.2 (Supplementary Table 1). In addition, one item was removed because of the distractor efficiency analysis. Considering all indexes, 30 items (17 knowledge and 13 practice) were removed from the administered 90-item questionnaire (Supplementary Table 1).

**Table 1:** Socio-demographic characteristics and working experience of participants (*N* = 154).

Variable		*N* (%)
Sex	Female	148 (96.1)
	Male	6 (3.9)
Age category	20-29 years	68 (44.1)
	30-39 years	43 (27.9)
	40-49 years	33 (21.4)
	50-59 years	10 (6.5)
Country of work	United Kingdom	124 (80.5)
	Other	30 (19.5)
Workplace	Hospital/clinical setting	58 (37.7)
	Community/primary care	16 (10.4)
	Freelance/ private practice	22 (14.3)
	Academia/ research	8 (5.2)
	Student dietitian	48 (31.2)
	Other	2 (1.3)
*Have you worked with IBS patients?*	Yes	108 (70.1)
	No	46 (29.9)
*How many years have you worked with IBS patients?*	None	44 (28.6)
	0-4 years	66 (42.2)
	5-9 years	24 (15.6)
	>10 years	21 (13.6)
Perceived experience in IBS dietary management (*n* = 1 missing)	Low (rating from 0 to 4)	57 (46.8)
	Moderate (ratings from 5 to 7)	59 (29.9)
	High (rating from 8 to 10)	37 (22.7)
Perceived experience of the low FODMAP diet (*n* = 1 missing)	Low (rating from 0 to 4)	72 (46.8)
	Moderate (ratings from 5 to 7)	46 (29.9)
	High (rating from 8 to 10)	35 (22.7)
Post-registration training on the low FODMAP diet	No	83 (53.9)
	Yes	51 (33.1)
	Currently undertaking a course	20 (13.0)

**Fig. 1:**
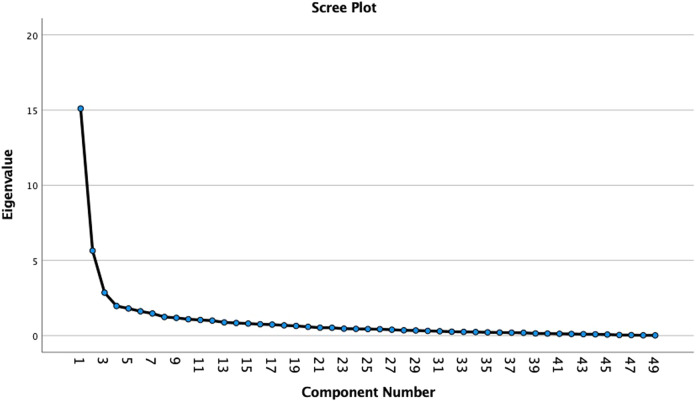
Scree plot from principal component analysis of the set of items. The eigenvalues are shown on the y-axis and the number of factors on the x-axis.

The KMO value was 0.88 and Bartlett’s test of Sphericity was χ^2^ = 6.040,6, df = 1176, *P* < 0.001, fulfilling the criteria for performing exploratory factor analysis. Principal component analysis with varimax rotation was performed and six factors were extracted based on the scree plot and eigenvalues >1 explaining 59.2% of the total variance (Fig. 1). All factors’ loadings were ≥0.4 and no items were cross-loaded. Factor 1 contained 13 items on low FODMAP diet counselling; Factor 2 contained 18 items on FODMAPs and gut health; Factor 3 contained 7 items on diagnosis and management of functional gut symptoms; Factor 4 contained 5 items on first-line dietary counselling; Factor 5 contained 3 items on attitudes towards the use of probiotics and Factor 6 contained 3 items on attitudes towards the implementation of the low FODMAP diet (Table 2). Confirmatory factor analysis found that the six-factor model was acceptable with a model χ2/df = 2.11, CFI = 0.97, TLI = 0.96, RMSEA = 0.08 and SRMR = 0.05. Out of 15 possible inter-factor correlations, 12 were significant (Table 3).

**Table 2:** Factors with loadings after principal component analysis with varimax rotation.

No	Item	Factors					
		1	2	3	4	5	6
Factor 1. Low FODMAP diet counselling							
P74	The definition and mechanisms of irritable bowel syndrome	0.87					
P76	The definition and role of visceral hypersensitivity in irritable bowel syndrome	0.76					
P77	The function of the gut-brain axis and its’ potential role in irritable bowel syndrome	0.79					
P78	The mechanisms with which FODMAPs trigger symptoms in irritable bowel syndrome	0.81					
P79	Foods high and low in FODMAPs	0.91					
P80	Preparation and cooking of low FODMAP meals	0.90					
P81	Food labelling	0.86					
P82	Challenges with shopping	0.91					
P84	Suitable options when eating out	0.86					
P85	Resources with foods high and low in FODMAPs	0.90					
P86	Cookbooks or resources with recipes	0.88					
P88	Use of a mobile app	0.83					
P89	Use of websites	0.83					
Factor 2. FODMAPs and gut health							
K10	Subtypes of irritable bowel syndrome according to the Rome IV criteria		0.42				
K18	Part of the gut associated with the highest microbial density and diversity		0.44				
K19	Food sources of probiotics or prebiotics (garlic)		0.40				
K25	Fructans may generate irritable bowel syndrome symptoms by decreasing stomach emptying		0.44				
K26	Fructans may generate irritable bowel syndrome symptoms by increasing colonic gas		0.52				
K27	Polyols may generate irritable bowel syndrome symptoms by increasing small intestinal water		0.40				
K28	Polyols may generate irritable bowel syndrome symptoms by increasing oesophageal sphincter relaxation		0.45				
K29	In irritable bowel syndrome, following a low FODMAP diet increases luminal bifidobacterial levels		0.45				
K31	Carbohydrate that assists the transport of fructose across the gastrointestinal mucosa		0.41				
K32	Source of FODMAPs (rye flour)		0.80				
K33	Source of FODMAPs (mango)		0.59				
K34	Source of FODMAP (onion)		0.75				
K35	Source of FODMAP (garlic)		0.75				
K36	Source of FODMAP (avocado)		0.48				
K38	Sources of FODMAPs (tempeh)		0.51				
K39	Sources of FODMAPs (honey)		0.59				
K40	Sweeteners low in FODMAPs		0.55				
A54	I would recommend a low FODMAP diet in patients with inflammatory bowel disease in remission and functional gastrointestinal symptoms		0.48				
Factor 3. Diagnosis and management of functional gut symptoms							
K9	Type of stools characterised by separate hard lumps like nuts according to the Bristol Stool Form Scale			0.69			
K11	Clinical tests recommended as part of the diagnosis of irritable bowel syndrome in the absence of red flag symptoms			0.76			
K13	Recommendation of drug Loperamide in the management of irritable bowel syndrome			0.65			
K24	Eating high FODMAP foods damages the gut lining and increases the risk of bowel cancer			0.57			
K44	All gluten free foods are low in FODMAPs			0.50			
A53	I would recommend a low FODMAP diet as a primary treatment in patients with active inflammatory bowel disease^a^			0.62			
A55	I would recommend a low FODMAP diet in patients with coeliac disease without functional gastrointestinal symptoms^a^			0.65			
Factor 4. First-line dietary counselling							
P61	Reduce caffeine intake if in excess				0.69		
P62	Reduce intake of high-fat foods, if in excess				0.64		
P66	Ensure dietary fibre intake is adequate				0.64		
P67	Ensure fruit and vegetable intake is adequate				0.65		
P72	Use wheat bran supplementation^a^				0.51		
Factor 5. Attitudes towards the use of probiotics							
A48	Taking a probiotic is safe in irritable bowel syndrome					0.58	
A50	I feel confident to recommend a probiotic if individuals with irritable bowel syndrome wish to try one					0.78	
A51	I am aware of resources and evidence-based recommendations regarding the use of probiotics in irritable bowel syndrome					0.74	
Factor 6. Attitudes towards the implementation of the low FODMAP diet							
A52	I would recommend a low FODMAP diet in patients with ongoing irritable bowel syndrome symptoms who have tried first-line dietary advice						0.40
A56	I would not recommend a low FODMAP diet in patients with a history of bulimia or anorexia nervosa						0.76
A57	I would not recommend a low FODMAP diet in patients with unexplained weight loss						0.76

^a^Reverse scoring.

**Table 3:** Correlation matrix of the different factors and overall items for each domain.

Factors	Factor 1	Factor 2	Factor 3	Factor 4	Factor 5	Overall knowledge	Overall attitudes	Overall practices
Factor 2	0.44**							
Factor 3	0.22*	0.51**						
Factor 4	0.66**	0.28**	0.32**					
Factor 5	0.17*	0.32**	0.30**	0.28**				
Factor 6	0.09	0.20*	0.18*	0.02	0.16			
Overall knowledge							0.53**	0.43**
Overall attitudes								0.25*
Overall practices								

*P < 0.05; **P < 0.001.

All items had item-total correlations greater than 0.2 except for items K10, A52 and A54. After removing these items, internal reliability using KR20 or Cronbach’s alpha was found >0.7 for each factor and 0.90, 0.64 and 0.97 for overall knowledge, attitudes and practice items, respectively. For external reliability, 28 participants were included in the test-retest analysis, one further participant was excluded from the analysis due to having attended a diet and IBS training course. The ICC of sum scores was >0.6 for each factor and 0.92, 0.78 and 0.98 for overall knowledge, attitudes and practice items, respectively (Table 4).

**Table 4:** Internal and external reliability of the KAP questionnaire.

Factors		Number of items	Internal reliability (*n* = 154)	External reliability (*n* = 28)^a^
			Cronbach’s alpha or KR20	ICC^b^ (95% CI)
Factor 1	Low FODMAP diet counselling	13	0.98	0.97 (0.93–0.98)
Factor 2	FODMAPs and gut health	16	0.89	0.92 (0.83–0.96)
Factor 3	Diagnosis and management of functional gut symptoms	7	0.73	0.75 (0.46–0.89)
Factor 4	First-line dietary counselling	5	0.93	0.99 (0.97–0.99)
Factor 5	Attitudes on the use of probiotics	3	0.71	0.66 (0.27–0.84)
Factor 6	Attitudes towards the implementation of the low FODMAP diet	2	0.75	0.84 (0.66-0.93)
Overall				
	Knowledge	21	0.90	0.92 (0.83–0.96)
	Attitudes	7	0.64	0.78 (0.51–0.90)
	Practice	18	0.97	0.98 (0.96–0.99)

^a^Difference in time is two weeks.

^b^ICC intraclass correlation coefficient.

The differentiation by ‘known-group’ analysis showed significant differences in sum scores between participants with different levels of IBS experience for each factor as well as overall knowledge, attitudes and practice items (Table 5). The final questionnaire consists of 46 items (21 knowledge items, 7 attitudes items and 18 practice items) (see Supplementary Information 2).

**Table 5:** Construct validity determined by differences in sum scores among dietitians with different levels of experience in IBS (*N* = 154)^a^.

Factors	Maximum score	Median (IQR)			Statistical test (degrees of freedom)	*P*-value
		Level of IBS experience				
		Low^b^ (*n* = 81)	Moderate^c^ (*n* = 42)	High^d^ (*n* = 31)		
Low FODMAP diet counselling	52	29.00 (39.00)	44.00 (11.25)	46.00 (10.00)	44.13 (2)	<0.001
FODMAPs and gut health	16	6.00 (6.00)	10.00 (6.25)	14.00 (3.00)	49.60 (2)	<0.001
Diagnosis and management of functional gut symptoms	15	12.00 (3.50)	15.00 (3.25)	14.00 (3.00)	25.65 (2)	<0.001
First-line dietary counselling	20	17.00 (7.00)	18.00 (4.00)	19.00 (3.00)	13.24 (2)	0.001
Attitudes towards the use of probiotics	15	12.00 (4.00)	12.00 (4.00)	13.00 (3.00)	7.82 (2)	0.020
Attitudes towards the implementation of the low FODMAP diet	10	9.00 (3.00)	8.00 (4.00)	10.00 (2.00)	6.81 (2)	0.033
*Overall*						
Knowledge	21	9.00 (7.00)	14.00 (8.25)	18.00 (3.00)	51.31 (2)	<0.001
Attitudes	35	27.00 (6.00)	28.50 (8.25)	31.00 (3.00)	11.29 (2)	0.004
Practices	72	47.00 (43.00)	61.00 (11.50)	65.00 (11.00)	44.57 (2)	<0.001

^a^The Kruskal–Wallis H test was used to determine any significant differences between the sum scores of each factor and overall sections among dietitians with low, moderate and high level of experience in IBS.

^b^Low: no experience of IBS or with ≤four years post-registration experience of IBS and no post-registration training (i.e. on the low FODMAP diet).

^c^Moderate: with ≤four years post-registration experience of IBS and undertaking/attended post-registration training or >four years post-registration experience of IBS without training or enrolled/undertaking post-registration training.

^d^High: with >5 years of experience of IBS and post-registration training.

## Discussion

A 46-item self-administered questionnaire has been developed and validated. Similar questionnaires exist assessing clinicians’ [30], students’ [31] and individuals’ [32, 33] knowledge, attitudes and practices in other areas. To the best of our knowledge, this is the first questionnaire to assess knowledge, attitudes and practices of UK-based dietitians in the dietary management of IBS. Due to the multifaceted approach of IBS management, the complexity of the low FODMAP diet and inter-individual variability, the use of this questionnaire in clinical practice requires additional assessment of dietitians’ communication skills [34] and individuals’ characteristics to ensure high-quality and patient-centred care in IBS management.

In this study, construct validity was assessed via exploratory factor analysis and the known-groups methods. The two methods were able to identify an acceptable model fit and significant differences in the performance of dietitians according to their level of experience in IBS management, demonstrating that the questionnaire has a good construct validity. It should be noted though that partial confirmatory analysis was applied providing a hypothetical structure of the questionnaire and the implementation of additional validity tests (e.g., criterion validity) would have further enhanced the overall validity of the questionnaire [17, 26]. Six latent constructs were found of which three addressed questions on the diagnosis and management of IBS including the use of probiotics and first-line dietary advice. The remaining three constructs addressed questions on FODMAPs and the implementation of the low FODMAP diet.

The questionnaire demonstrated very good reliability with an internal consistency greater than 0.7 (Cronbach’s alpha) in most factors and overall items. It is important to note though that sample size and the dimensionality of the questionnaire can affect internal consistency coefficients with many arguing that Cronbach’s alpha should be reported for items under the same construct (factor) and not for overall items [35]. In this questionnaire, internal consistency was calculated for each factor and was greater than that of all items (e.g., attitudes domain) enhancing the internal consistency of the tool. External consistency was assessed by calculating the ICC of sum scores across time and all factors and overall items for each domain demonstrated an external consistency greater than 0.7 (except factor 5). An interval of two weeks was considered a sufficient time to assess whether participants’ performance was repeatable and prevent recall biases or any significant increases in participants’ performance.

Although the initial questionnaire included a wide range of questions related to IBS, the final questionnaire is skewed towards a higher number of FODMAP-related questions. In part this may be due to two thirds of participants (70.1%) having had some education and even clinical experience of IBS (i.e., post-registration) and only one third having had no experience (i.e. pre-registration). Even newly appointed gastroenterology dietitians are expected to be familiar with basic facts and practices in IBS diagnosis and management, thus the performance of many items did not significantly differ between participants, resulting in the removal of these items. On the other hand, only 22.7% of participants reported having high experience with the low FODMAP diet and only 13% had received post-graduation training. This resulted in greater distinguishment between low and high performers during the validation process and may explain the inclusion of more FODMAP-related questions in the final questionnaire. It also confirms the need for more dietitians to be trained to deliver the low FODMAP diet [12].

The applicability of the questionnaire in other healthcare professionals as well as outside of the UK needs to be further explored. Many healthcare professionals offer first line dietary advice before referring patients to dietitians [36] while others recommend the low FODMAP, despite lacking the appropriate level of knowledge and skills to communicate the diet [37]. Testing this questionnaire among healthcare professionals other than dietitians is warranted to identify if the questionnaire can be used to assess their knowledge and competence in the provision of dietary advice in IBS and to determine the type and level of information dietitians and other healthcare professionals should deliver to IBS patients. Most participants (80.5%) were UK-based and many items were developed in line with the current UK guidelines [6] and resources available in the UK [38]. Thus, further adaptations and validation tests are needed to ensure the applicability of the questionnaire outside of the UK.

Limitations of the study include the small sample size, as many argue that samples sizes of 300 or more are needed for questionnaire development and validation [17]. The sample size of this study was considered sufficient though and fulfilled the criteria to perform the appropriate statistical tests (e.g., factor analyses, test-rest reliability) for the questionnaire validation. The advertisement of the questionnaire via social media provides limited information on how many dietitians received the invitation for the study and chose not to participate. The administration of the questionnaire online can also increase the chances of searching for the correct answers to the knowledge items. Regarding the multiple-choice items, participants had the option of choosing ‘not sure’ rather than guessing. The ‘not sure’ option was used to avoid guessing, however, it may also prevent participants from thinking the right option. In addition, many students chose the option of ‘neither agree nor disagree’ for the attitude items and the ‘not applicable’ option for the practice items which may explain the weak correlation found between attitudes and practices (*r* = 0.25) in the questionnaire. For content validity only written feedback was provided and for face validity only one focus group took place due to many dietitians being redeployed during the Covid-19 pandemic. However, the team reviewed the feedback received from both experts and practice dietitians and considered it adequate to make amendments and administer the refined draft of the questionnaire to the study population for psychometric testing.

In conclusion, the final 46-item questionnaire is an easy-to-use self-administered tool assessing knowledge, attitudes and practices of UK-based dietitians in IBS dietary management with higher scores indicating greater performance in each domain. It is a newly developed questionnaire with strong reliability and validity. It can be used in clinical and public health practice to investigate gaps in knowledge and training needs of staff as well as in research to assess the efficacy of interventions aiming to increase knowledge and competence of clinicians or investigate the relationship of clinicians’ knowledge, attitudes and practices with patients’ outcomes in irritable bowel syndrome.

## Supplementary information

Item reduction analysis of the set of items after the survey administration

Knowledge, Attitudes and Practices Questionnaire in the dietary management of irritable bowel syndrome

## Data Availability

The datasets generated and analysed during the current study are available from the corresponding author on reasonable request.
