# Supplementary material for: Development and validation of a knowledge, attitudes and practices questionnaire in the dietary management of irritable bowel syndrome
Source: Eur J Clin Nutr. 2023 Jul 12;77(9):911–8. doi: 10.1038/s41430-023-01306-7 (PMC10473958; doi:10.1038/s41430-023-01306-7)
Supplement: Supplementary file 1 — Item reduction analysis of the set of items after the survey administration [file 41430_2023_1306_MOESM1_ESM.pdf]

Supplementary Information Table 1. Item reduction analysis of the set of items after the survey administration

No	Item	Item discrimination[a]	Item difficulty[b]	Item validity [c]	Item-total correlation[d]
		D	*P*	*Point-biserial r*	*r*
	Knowledge				
1	IBS is more prevalent in men than women⊗	0.12	0.81	0.11	
2	IBS is more prevalent in older people	0.43	0.68	0.40	
3	IBS is more prevalent in developed countries⊗	0.31	0.73	0.28	
4	Aetiopathogenesis of IBS⊗	0.24	0.93	0.31	
5	Factors associated with IBS⊗	0.17	0.93	0.30	
6	Description of visceral hypersensitivity	0.62	0.73	0.55	
7	NOT a known symptom of functional bowel disorders⊗[e]	0.36	0.85	0.38	
8	Rome IV diagnostic criteria⊗	0.26	0.81	0.33	
9	The Bristol Stool Form Scale and type of stools	0.40	0.80	0.41	
10	Subtypes of IBS	0.36	0.37	0.35	
11	Clinical tests diagnosis of IBS	0.48	0.67	0.41	
12	Assessment of response to dietary intervention in IBS⊗	0.17	0.91	0.22	
13	Use of drug Loperamide	0.60	0.68	0.52	
14	The colonic luminal microbiota metabolise fermentable carbohydrates producing short chain fatty acids and gas⊗	0.31	0.85	0.36	
15	The mucosal microbiota regulates immune homeostasis⊗	0.31	0.55	0.25	
16	Probiotics are live microorganisms that when ingested in adequate amounts. exert a health benefit to the host⊗	0.12	0.91	0.22	
17	Prebiotics are fermentable carbohydrates that diminish the selective growth of beneficial bacteria in the gut⊗	0.36	0.71	0.27	
18	Part of the gut is associated with the highest microbial density and diversity	0.36	0.76	0.40	
19	Food sources of probiotics or prebiotics_ Garlic	0.45	0.67	0.37	
20	Food sources of probiotics or prebiotics_Kefir⊗	0.12	0.73	0.12	
21	Food sources of probiotics or prebiotics_Chicory⊗	0.33	0.60	0.26	
22	Food sources of probiotics or prebiotics_Cheddar cheese	0.48	0.55	0.36	
23	FODMAPs may generate IBS symptoms by increasing small intestinal water and colonic bacterial fermentation	0.5	0.79	0.50	
24	Eating high FODMAP foods damages the gut lining and increases the risk of bowel cancer	0.4	0.88	0.40	
25	Fructans may generate IBS symptoms by decreasing stomach emptying	0.9	0.42	0.64	
26	Fructans may generate IBS symptoms by increasing colonic gas	0.5	0.78	0.50	
27	Polyols may generate IBS by increasing small intestinal water	0.7	0.56	0.53	
28	Polyols may generate IBS by increasing oesophageal sphincter relaxation	0.8	0.47	0.58	

29	In IBS, following a low FODMAP diet increases luminal bifidobacterial levels	0.8	0.41	0.59	
30	Nutrients reduced when following a low FODMAP diet	0.7	0.51	0.49	
31	Carbohydrate that assists the transport of fructose across the gastrointestinal mucosa	0.6	0.55	0.43	
32	Source of FODMAPs_Rye flour	0.9	0.46	0.69	
33	Source of FODMAPs_Mango	0.6	0.61	0.43	
34	Source of FODMAP_Onion	0.8	0.49	0.57	
35	Source of FODMAP_Garlic	0.8	0.48	0.60	
36	Source of FODMAP_Avocado	0.7	0.31	0.55	
37	Source of FODMAPs_Soya milk⊗	0.5	0.22	0.39	
38	Sources of FODMAPs_Tempeh	0.6	0.31	0.45	
39	Sources of FODMAPs_Honey	0.7	0.58	0.51	
40	Sweeteners low in FODMAPs	0.6	0.62	0.45	
41	Most soft and hard cheeses are naturally low in lactose and therefore can be included during FODMAP restriction	0.5	0.51	0.34	
42	Green beans are high in FODMAPs and should be avoided during FODMAP restriction	0.6	0.45	0.45	
43	All fruit is high in fructose and should be avoided during FODMAP restriction⊗	0.4	0.79	0.31	
44	All gluten free foods are low in FODMAPs	0.5	0.75	0.46	
45	Lactose free milk is a safe alternative to cow’s milk during FODMAP restriction⊗	0.5	0.82	0.37	
46	The reintroduction stage enables patients to use food challenges for high FODMAP foods in increasing portion size to ascertain an individual's tolerance level⊗	0.3	0.88	0.40	
47	At the end of FODMAP reintroduction all high FODMAP foods can be added back to the patient’s diet	0.5	0.74	0.40	
	Attitudes				
48	Taking a probiotic is safe in irritable bowel syndrome				0.38
49	Taking a prebiotic relieves diarrhoea in IBS				0.20
50	I feel confident to recommend a probiotic if individuals with IBS wish to try one				0.36
51	I am aware of resources and evidence-based recommendations regarding the use of probiotics in IBS				0.43
52	I would recommend a low FODMAP diet in patients with ongoing IBS symptoms who have tried first-line dietary advice				0.28
53	I would recommend a low FODMAP diet as a primary treatment in patients with active inflammatory bowel disease.				0.29
54	I would recommend a low FODMAP diet in patients with inflammatory bowel disease in remission and functional gastrointestinal symptoms				0.26
55	I would recommend a low FODMAP diet in patients with coeliac disease without functional gastrointestinal symptoms				0.38
56	I would not recommend a low FODMAP diet in patients with a history of bulimia or anorexia nervosa				0.30

57	I would not recommend a low FODMAP diet in patients with unexplained weight loss				0.40
	Practice				
58	Which of the following dietary modifications are NOT included in first-line dietary advice for irritable bowel syndrome?- a gluten free diet⊗				0.12
59	Which of the following are recommended in the management of irritable bowel syndrome?- peppermint oil⊗				-0.04
60	Elimination diets are appropriate for first-line dietary treatment of IBS				0.34
61	Reduce caffeine intake if in excess				0.45
62	Reduce intake of high-fat foods, if in excess				0.48
63	A low lactose diet if lactose intolerance is suspected⊗				-0.30
64	A gluten-free diet⊗				-0.33
65	Drink adequate fluids⊗				-0.39
66	Ensure dietary fibre intake is adequate				0.56
67	Ensure fruit and vegetable intake is adequate				0.52
68	Increase oat intake⊗				-0.27
69	Increase kiwi consumption⊗				-0.28
70	Increase prune consumption⊗				-0.49
71	Use linseed supplementation⊗				-0.29
72	Use wheat bran supplementation				0.46
73	How many weeks do you advise a patient with IBS to follow the restriction stage of the low FODMAP diet? 4-8 weeks				0.35
74	The definition and mechanisms of IBS				0.80
75	The definition of functional gut disorders⊗				-0.48
76	The definition and role of visceral hypersensitivity in IBS				0.75
77	The function of the gut-brain axis and its' potential role in IBS				0.72
78	The mechanisms with which FODMAPs trigger symptoms in IBS				0.80
79	Foods high and low in FODMAPs				0.87
80	Preparation and cooking of low FODMAP meals				0.88
81	Food labelling				0.81
82	Challenges with shopping				0.86
83	Cost of low FODMAP foods⊗				-0.44
84	Suitable options when eating out				0.80
85	Resources with foods high and low in FODMAPs				0.84
86	Cookbooks or resources with recipes				0.86
87	Written diet sheets or adaptations to an existing food diary⊗				-0.45
88	A mobile app				0.80
89	Websites				0.78
90	Online social groups⊗				-0.56

[a] Calculated using Kelley's formula. Acceptable values >0.4

[b] Very easy (>0.7) or very difficult (<0.3) items were excluded

[C] Pearson correlation. Acceptable values r>0.4

[d] Corrected item-total correlations. Acceptable values ≥0.2

[e] Item removed due to distractor efficiency analysis

⊗ Items removed
