# Supplementary material for: Development and validation of a knowledge, attitudes and practices questionnaire in the dietary management of irritable bowel syndrome
Source: Eur J Clin Nutr. 2023 Jul 12;77(9):911–8. doi: 10.1038/s41430-023-01306-7 (PMC10473958; doi:10.1038/s41430-023-01306-7)
Supplement: Supplementary file 2 — Knowledge, Attitudes and Practices Questionnaire in the dietary management of irritable bowel syndrome [file 41430_2023_1306_MOESM2_ESM.pdf]

Knowledge, Attitudes and Practices Questionnaire in the dietary management of irritable bowel syndrome

Introduction

This is a self-administered questionnaire for dietitians to assess Knowledge, Attitudes and Practices of irritable bowel syndrome.

The questionnaire includes three main sections. The first section includes knowledge questions in relation to the diagnosis and management of functional gut symptoms followed by questions on FODMAPs. FODMAPs stand for fermentable oligosaccharides (fructans and a-galacto-oligosaccharides), disaccharides, monosaccharides and polyols. The second section is about attitudes towards the use of probiotics and the practice of the low FODMAP diet. The third section is about practices of first-line dietary advice and consultation of the low FODMAP diet in the management of irritable bowel syndrome.

If you do not know the answer in any of the questions, please mark “not sure” rather than guess. If you have never worked with patients with irritable bowel syndrome, choose “not applicable” when relevant.

The first questions are about the diagnosis and management of functional gut symptoms

1. Which of the following clinical tests are recommended as part of the diagnosis of irritable bowel syndrome in the absence of red flag symptoms (e.g., bleeding, unexplained weight loss)?

    - Lactulose breath test
    - **Coeliac antibodies**
    - Colonoscopy
    - Not sure

2. The Bristol Stool Form Scale is used to characterise the consistency of stool. Which type of stool is characterised by separate hard lumps, like nuts?

    - **Type 1**
    - Type 2
    - Type 3
    - Not sure

3. Which of the following would the drug Loperamide be a good choice in the management of irritable bowel syndrome?

    - Abdominal pain
    - Constipation
    - **Diarrhoea**
    - Not sure

4. A gluten-free diet is part of the first-line dietary advice for irritable bowel syndrome.

    - True
    - **False**
    - Not sure

The next questions are about gut health and FODMAPs

5. Which part of the gut is associated with the highest microbial density and diversity?
    - Stomach
    - Small intestine
    - **Colon**
    - Not sure
6. Which carbohydrate assists the transport of fructose across the gastrointestinal mucosa?
    - **Glucose**
    - Sucrose
    - Lactose
    - Not sure

Choose whether the following statements are true or false

	True	False	Not sure
7. Fructans may generate symptoms in irritable bowel syndrome by decreasing stomach emptying.		v	
8. Fructans may generate symptoms in irritable bowel syndrome by increasing colonic gas.	v		
9. Polyols may generate symptoms in irritable bowel syndrome by increasing oesophageal sphincter relaxation.		v	
10. Polyols may generate symptoms in irritable bowel syndrome by increasing small intestinal water.	v		
11. Eating low FODMAP foods in irritable bowel syndrome increases luminal bifidobacterial levels.		v	
12. Eating high FODMAP foods in irritable bowel syndrome can damage the gut lining and increase the risk of bowel cancer.		v	

13. Which of the following is a good source of prebiotics?

- Kefir
- **Garlic**
- Cheddar cheese
- Not sure

Choose which FODMAPs are present in high amounts in each of the following foods (choose only one option)

	Fructans	Galacto-oligosaccharides	Fructose	Polylos	Not high in FODMAPs	Not sure
14. Rye flour	v					
15. Mango			v			
16. Garlic	v					
17. Onion	v					
18. Avocado				v		
19. Honey			v			
20. Tempeh					v	

21. Which of the following sweeteners are low in FODMAPs?

- Xylitol
- **Stevia**
- Mannitol
- Not sure

The following section is about attitudes in irritable bowel syndrome management

Please rate your agreement with each of the following statements about the use of probiotics in the management of irritable bowel syndrome.

	Strongly disagree	Somewhat disagree	Neither agree nor disagree	Somewhat agree	Strongly agree
22. Taking a probiotic is safe in irritable bowel syndrome.					
23. I feel confident to recommend a probiotic, if individuals with irritable bowel syndrome wish to try one.					
24. I am aware of resources and evidence-based recommendations regarding the use of probiotics in irritable bowel syndrome.					

Please rate your agreement with each of the following statements in relation to the practice of the low FODMAP diet.

	Strongly disagree	Somewhat disagree	Neither agree nor disagree	Somewhat agree	Strongly agree
25. I would recommend a low FODMAP diet in patients with coeliac disease without functional gastrointestinal symptoms.*					
26. I would recommend a low FODMAP diet as a primary treatment in patients with active inflammatory bowel disease.*					
27. I would not recommend a low FODMAP diet in patients with a history of bulimia or anorexia nervosa.					
28. I would not recommend a low FODMAP diet in patients with unexplained weight loss.					

*reverse scoring

The following section is about practices in irritable bowel syndrome management

Please rate how often you recommend the following first-line dietary advice to an individual with irritable bowel syndrome.

	Never	Sometimes	Often	Always	Not applicable
29. Reduce caffeine intake, if in excess.					
30. Reduce intake of high-fat foods, if in excess.					
31. Ensure fruit and vegetable intake is adequate.					
32. Ensure dietary fibre intake is adequate.					
33. Use wheat bran supplementation.*					

*reverse scoring

Please rate how often you recommend or practice the following during a low fodmap diet consultation.

	Never	Sometimes	Often	Always	Not applicable
34. Explain the definition and mechanisms of irritable bowel syndrome.					
35. Explain the definition and role of visceral hypersensitivity in irritable bowel syndrome.					
36. Explain the function of the gut-brain axis and its potential role in irritable bowel syndrome.					
37. Explain the mechanisms with which FODMAPs trigger symptoms in irritable bowel syndrome.					
38. Educate about foods high and low in FODMAPs.					
39. Discuss challenges with shopping.					
40. Discuss suitable options when eating out.					
41. Discuss the preparation and cooking of low FODMAP meals.					
42. Provide resources with foods high and low in FODMAPs.					
43. Recommend cookbooks or recipes.					
44. Educate on food labelling.					
45. Recommend a mobile app.					
46. Recommend websites.					

Thank you for your time
